# Best Mode of Storing Ice

**Published:** 1885-12

**Authors:** 


### BEST MODE OF STORING ICE.

If one will need ice next summer, he must prepare for it now. The first thing to be done is to gather a few wagon loads of sawdust—about seven hundred bushels will be required for a house twelve feet square, and ten feet high, to give an ample supply. If sawdust can not be procured, dry waste tan-bark is equally good ; dry swamp muck, forest leaves, cut straw chaff, or chaff from the threshing machine, are all very good substitutes ; but an open air space is only about forty per cent. as effective as any one of these substances. A house twelve feet square will hold a mass of ice ten feet square, which will give about five thousand pounds for each foot in height, yielding a supply of one hundred pounds daily, for about two months. One hnndred pounds of ice will cool one hundred pounds of water from one hundred and seventy-four degrees, down to thirty-two degrees, absorbing one hundred and forty-two degrees of heat from the water, in the slow process of liquefaction alone. These figures will enable any person to calculate how much ice may be required for any specified effect. Thus as one hundred pounds of ice, absorbs fourteen thousand and two hundred units of heat, and we want to cool seven hundred and ten pounds of milk from sixty-five to forty-five degrees, we shall find that the ice will just do it, because seven hundred and ten pounds cooled twenty-eight degrees, equals fourteen thousands and two hundred units. In the use of ice, it is therefore seen to be a great economy, to cool the milk down to just as low a point as possible, by means of cold well or spring water, before it is set in the ice water pool. For a three hundred quart dairy, or for twenty cows, then, one hundred pounds of ice will be required daily, and for the season of eight months, when ice may be necessary, the ten feet square of ice should be raised eight feet, which will allow for waste, which is usually about forty or fifty per cent on the average.—*American Agricultural for November.*

